# Prevalence, barriers and factors associated with parental disclosure of their HIV positive status to children: a cross-sectional study in an urban clinic in Kampala, Uganda

**DOI:** 10.1186/s12889-016-3235-2

**Published:** 2016-07-11

**Authors:** Charles Peter Osingada, Monica Okuga, Rose Chalo Nabirye, Nelson Kaulukusi Sewankambo, Damalie Nakanjako

**Affiliations:** Department of Nursing, Makerere University College of Health Sciences, School of Health Sciences, P.O. Box 7072, Kampala, Uganda; Department of Health Policy planning and Management, Makerere University School of Public Health, P.O. Box 7072, Kampala, Uganda; Department of Internal Medicine, School of Medicine, Makerere College of Health Sciences, P.O. Box 7072, Kampala, Uganda

**Keywords:** HIV Disclosure, Prevalence, Barriers, Associated factors, Uganda

## Abstract

**Background:**

Disclosure of parental HIV status is associated with a number of positive outcomes such as improved adherence to clinic appointments, lower levels of parental anxiety and depression, and mutual emotional support between parents and their children. Very few studies in low-resource settings have addressed the issues of parental disclosure of their HIV status to their children.

**Methods:**

A cross-sectional study was conducted among adult parents attending HIV/AIDS prevention, care and treatment clinic at Makerere University Infectious Diseases Institute (IDI), Kampala, Uganda. Participants were interviewed using the Parent Disclosure Interview (PDI) questionnaire which is a standard tool developed specifically for HIV infected parents. Data were analyzed using STATA version 13.1.

**Results:**

Of 344 participants, only 37 % had told at least one of their children that they were HIV positive. Barriers to disclosure were fear that children may tell other people about the parent’s HIV status, desire not to worry or upset children and perceptions that children may not understand. Age of the parent, religion and having someone committed to care of the children were positively associated with parental disclosure of their HIV positives status. Attainment of tertiary level of education was negatively associated with parental disclosure of their HIV status.

**Conclusions:**

Parental disclosure of a positive HIVstatus to their children is still low in urban Kampala. There is therefore need to develop locally relevant interventions so as to increase rates of parental disclosure of a positive HIV status to their children and thus promote open and honest discussions about HIV/AIDS at family level.

**Electronic supplementary material:**

The online version of this article (doi:10.1186/s12889-016-3235-2) contains supplementary material, which is available to authorized users.

## Background

Globally, it is estimated that by the end of 2013, there were 35 million people living with HIV and about 70 % of these live in sub-Saharan Africa, a region hardest hit by the epidemic. In Uganda approximately 7.3 % of adults aged 15–49 years are estimated to be living with HIV as of 2011 [1]. Wide spread roll-out and increasing availability of Antiretroviral therapy (ART) have effectively transformed HIV infection into a chronic condition. Thus, many HIV positive individuals live longer and are able to support their families and even have more children. Findings from other studies indicate that parents struggle with the issues of disclosure of their HIV status to their children and some choose not to do it all together for fear of negative consequences such as stigma, abandonment, and rejection [2–4]. However, parental disclosure of their HIV positive status to their children is associated with benefits for both the children and parents. In some USA studies it has been reported that problem behaviors decreased over time among children who had been disclosed to compared to those who had not been disclosed to [5]. Children who had been disclosed to by their mothers displayed lower levels of aggressiveness and negative self-esteem than children who had not been disclosed to [6]. Also children who had been disclosed to by their parents exhibited a higher self-concept over time [7].

For the case of parents, parental disclosure of their HIV status to their children has been found to be critical in promoting psychological and social well being of the children and parents. Studies indicate that parental disclosure to children is associated with improved parental adherence to clinic appointments [8], lower levels of anxiety and depression and better mutual emotional [9] and social support [4, 6]. Furthermore, family cohesion is reported to be significantly higher among disclosers than non-disclosers [10].

Compared to the situation in developed countries, in low resource countries, little attention has been directed to parental disclosure of their own HIV positive status to children. This has just recently attracted attention of researchers and program implementers even though developing countries particularly in sub-Saharan Africa continue to bear high rates of HIV infection [11]. In Uganda, little is known about the prevalence and factors associated with parental positive HIV disclosure to their children. This study therefore sought to determine the prevalence and factors associated with parental disclosure of their HIV positive status to their children with the aim of informing policy and practice.

## Methods

### Study design and setting

This was a cross-sectional study which employed quantitative data collection methods. The study was conducted at the Infectious Diseases Institute (IDI), Makerere University, Uganda. The Infectious Diseases Institute is a Ugandan not-for-profit organization within Makerere University whose mission is to strengthen health systems in Africa, with a strong emphasis on infectious diseases, through research and capacity development. Established within Makerere University, the Institute began in 2002 and currently provides care and treatment services to over 110,000 people living with HIV in urban and rural settings in Uganda- directly through a large outpatient’s clinic, and in partnership with government and non-government health facilities, which amounts to about 13 % of the national effort. The institute also provides extensive prevention services (including medical male circumcision) and is a national referral centre for complicated cases of HIV [12]. The IDI clinic located at Mulago hill, just adjacent to Mulago national referral hospital, currently provides care and treatment to 8,025 active clients with an average of 308 clients seen per day. This site was chosen because it has a prevention, care and treatment clinic with the largest number of HIV positive clients in urban Kampala.

### Sample size calculation

The sample size was estimated using the formula z^2^pq/d^2^. Assuming a prevalence of parental disclosure to be 50 %, 95 % confidence interval z-value of 1.96 and 5 % margin of error, the required sample size was calculated as 384 participants. The 50 % used in calculating the sample size was based on results from a qualitative study conducted by Rwemisisi and colleagues [4]. During data analysis it was noted that of the 384 participants recruited, 344 had children thus 40 participants did not have children and were erroneously included in the study.

### Participant recruitment and data collection procedures

The study participants comprised HIV positive parents aged 18 years and older and were attending the IDI clinic at Mulago. Parents who were HIV positive, had child/children aged 5 years or older and consented were included in the study. Parents who were very sick or mentally incapable of proving informed consent were excluded from the study. Participant for the study were drawn from IDI Mulago clinic. The clinic provides a range of health services to HIV positive individuals on a daily basis. Participants for the study were consecutively recruited from among patients attending the outpatient clinic. On every clinic day the research team screened all clients for eligibility to participate in the study. In order not to disrupt smooth running of the clinic, parents who met the recruitment criteria and accepted to participate in the study were given appointments on when to return to the clinic for the interviews. On the day of the interview, each parent was taken through the consenting process and those who provided their written informed consent were interviewed by a trained research assistant. Each interview took about forty five minutes and all the interviews were conducted in Luganda (local language spoken in central Uganda). To ensure privacy and confidentiality, each interview was conducted in a quiet room which is usually used for counseling clients.

Data were collected using the Parent Disclosure Interview (PDI) questionnaire [13]. The PDI is a standardized/validated questionnaire developed specifically for HIV infected parents and has been used in the USA but has never been used or validated in a Ugandan population. The tool was not locally validated but in a study conducted in the USA the tool was found to elicit reliable information with regard to disclosure of HIV to family members including adults and young children. The items in the tool have consistently “good” to “excellent” reliability coefficients [13]. The questionnaire was adapted and translated to Luganda by an expert from Makerere University Department of Linguistics, English language studies and communication. Back translation was done to check for consistence in meaning. The translated questionnaire was pre-tested among HIV positive parents attending Old Mulago general outpatient department. All necessary adjustments were made before data collection.

### Data entry and statistical analysis

Data was edited to check for consistency and data entry screen was designed using census and survey processing (CSPro) software version 6.0 with checks and skips for quality assurance. The data was entered by experienced data entrants. It was cleaned and analyzed by a statistician using STATA version 13.1. Means and standard deviations were computed for continuous data and frequencies, proportions for categorical data. Bivariate and multivariable analyses were conducted using logistic regression with manual backward stepwise regression in order to obtain the covariates that were associated with parental disclosure. The strength of the association between dependent and independent variables was determined using odds ratios, and the significance of the association was determined using confidence intervals. During adjusted analysis, variables found to be statistically significant at bivariate level and the insignificant variables which were deemed to be important in influencing the outcome variable, were entered into the logistic regression model. Factor analysis was conducted to identify important barriers towards parental disclosure of their HIV status to children.

## Results

### Socio-demographic characteristics of participants

About fifty three percent (205) of the respondents were female (Table 1). The mean age of the respondents was 42.4(SD 11.3) years. Ninety eight percent (378) of the respondents were using an HIV drug, that is, either Cotrimoxazole or antiretroviral drug or both.

**Table 1:** Demographic characteristics

Variable	Number	Percent (%)
Gender of respondent	*n* = 384	
Female	205	53.4
Male	179	46.6
Education	*n* = 384	
No education	16	4.2
Primary	189	49.2
Secondary	132	34.4
Tertiary	47	12.2
Religion	*n* = 384	
Protestant (COU)	95	24.7
Catholic	141	36.7
Pentecostal (born again)	86	22.4
Muslim	52	13.5
SDA	7	1.8
Others	3	0.8
Using any HIV drug	*n* = 384	
Yes	378	98.4
Type of Drug	*n* = 378	
ARVs	10	2.6
Septrin (Cotrimoxazole)	9	2.4
Both	359	95.0
Belong to HIV support group	*n* = 381	
Yes	25	6.6
Age	*n* = 384	
20 to 29	54	14.1
30 to 39	100	26
40 to 49	133	34.6
50 to 59	62	16.1
60 +	35	9.1
Mean age (SD)		42.4 (11.3)
Marital Status	*n* = 384	
Single	41	10.7
Married/cohabiting	200	52.1
Divorced/separated	83	21.6
Widowed	60	15.6
Told husband/wife about HIV infection	*n* = 200	
Yes	166	83.0
Anyone made a commitment to care for your child/ren		
Yes	121	85.8

### Prevalence of parental disclosure of their HIV positive status to their children

Of 344 parents with children only 37 % (127) had told at least one of their children that they were HIV positive. Of the 127 children who were informed about their parents HIV status, about 98 % (124) were informed more than a year ago. Forty nine percent (62) of the respondents who disclosed to at least one child know someone else who has disclosed his/her HIV positive status to their children. Table 2 shows the Parental disclosure of HIV positive status to the children.

**Table 2:** Parental disclosure of HIV positive status to children

Variable	Number	Percent (%)
Have children (own or adopted)	*n* = 384	
Yes	344	89.6
Told/Disclosed to at least one child about HIV Positive status	*n* = 344	
Yes	127	36.9
Timing when told the children	*n* = 127	
Less than a year ago	3	2.4
More than a year but less than 2 years	14	11.0
More than 2 years ago but less than 5 years	17	13.4
More than 5 years ago	15	11.8
No response	78	61.4
Told all children 18 or older about HIV infection	*n* = 127	
Yes	45	35.4
Know anyone else who has disclosed his HIV+ status to children	*n* = 127	
Yes	62	48.8

### Barriers to parental disclosure of their HIV status to their children

Table 3 shows that among the 217 parents who had not disclosed, 49.8 % (110) thought that their children may tell other people about their HIV infection, 54.8 % (121) did not want to worry or upset their children and 60.4 % (134) thought that the children may not understand what being HIV positive means.

**Table 3:** Barriers to parental disclosure of their HIV+ status to children

Item	Loading		Degree of importance			
	1	2	Not important	A little important	Important	Very important
Worry that the children will ask them how they got infected	0.559	0.310	143 (64.4)	12 (5.4)	27 (12.2)	40 (18.0)
Worry that the children will not care about them if they found out that their parent has AIDS	0.419	0.399	185 (83.7)	6 (2.7)	10 (4.5)	20 (9.1)
Their children may tell other people about their HIV infection	0.525	0.508	78 (35.3)	14 (6.3)	19 (8.6)	110 (49.8)
They do not want to worry or upset the children	0.840	−0.052	61 (27.6)	19 (8.6)	20 (9.1)	121 (54.8)
Concerned that they will have to talk to the children about death or dying	0.846	−0.090	99 (45.2)	18 (8.2)	28 (12.8)	74 (33.8)
The children may not understand it	−0.164	0.847	62 (27.9)	8 (3.6)	18 (8.1)	134 (60.4)

### Factors associated with parental disclosure of their HIV positive status to their children

Table 4 shows the determinants for parental disclosure or nondisclosure of their HIV positive status. According to the adjusted logistic regression analysis moving from a lower age category into a higher age category increased the odds of parents disclosing their HIV positive status to their children. Parents in the 30 to 39 years category were five times more likely to disclose as compared to those in the 20–29 years category (AOR 5.2, 95 % CI 2.0–13.1). Parents in the 40 to 49 years category were 7 times more likely to disclose their HIV positive status to their children compared to those in 20 to 29 years category (AOR 7.8, 95 % CI 3.0–20.7) and parents in 50 to 59 years category were eleven times more likely to disclose to their children their HIV positive status as compared to the parents in the 20 to 29 years category (AOR 11.3, 95 % CI 3.2–40.2) and parents who were above 60 years were also eleven times more likely to disclose their status compared to those in the 20 to 29 years category (AOR 11.7, 95 % CI 2.9–47.5). Parents who had obtained tertiary level of education were 9 times less likely to disclose their HIV positive status to their children as compared to the parents who did not have any education (AOR 0.1, 95 % CI 0.0–0.4). Parents who belonged to the Pentecostal(born again) faith were eight times more likely to disclose their HIV positive status to their children compared to parents who belonged to the protestant faith(AOR 8.0, 95 % CI 3.3–19.5). Parents who had someone committed to take care of the children were 2.5 times more likely to disclose their HIV status to their children as compared to those who did not (AOR 2.5, 95 % CI 1.1–5.7).

**Table 4:** Determinants of Parental disclosure

Variable	OR (95 % CI)	AOR (95 % CI)
Age		
20 to 29	1	1
30 to 39	2.7 (1.2–5.8)	5.2 (2.0–13.1)*
40 to 49	2.4 (1.1–5.1)	7.8 (3.0–20.7)*
50 to 59	2.9 (1.2–6.9)	11.3 (3.2–40.2)*
60 +	1.4 (0.5–4.3)	11.7 (2.9–47.5)*
Gender		
Male	1	
Female	1.0 (0.7–1.6)	1.4 (0.8–2.5)
Education		
No education	1	
Primary	1.1 (0.4–3.3)	0.4 (0.1–1.8)
Secondary	0.8 (0.3–2.5)	0.3 (0.1–1.3)
Tertiary	0.2 (0.0–0.8)	0.1 (0.0–0.4)*
Religion		
Protestant (COU)	1	
Catholic	1.0 (0.5–1.7)	1.1 (0.6–2.1)
Pentecostal (born again)	2.2 (1.1–4.1)	8.0 (3.3–19.5)*
Muslim	0.9 (0.4–1.8)	0.8 (0.4–1.7)
Others	0.6 (0.1–2.8)	(OR omitted)^¤^
Marital Status		
Single	1	
Married/cohabiting	1.5 (0.7–3.3)	
Divorced/separated	2.2 (0.9–5.2)	
Widowed	2.7 (1.1–6.7)	
Told husband/wife about HIV infection		
No	1	
Yes	9.6 (1.2–74.0)	
Anyone made a commitment to care for your children		
No	1	
Yes	3.3 (1.1–10.6)	2.5 (1.1–5.7)*

^¤^OR was omitted because in Table 3 the cell count ≤5 for both disclosure and none disclosure

*P < 0.05

## Discussion

### Prevalence of parental disclosure of their HIV status to children

In this study we found that only 37 % of the parents had disclosed their HIV positive status to at least one of their children. This figure is less than earlier findings from a qualitative study by Rwamesisi and colleagues [4] but slightly higher than 28 % [14] and 30 % [6] reported by studies conducted among HIV positive mothers in the United States of America. Even though our finding is slightly less than what was earlier reported from Uganda, it is important to note that to the best of our knowledge this study represents the first major effort in urban Kampala to investigate prevalence of parental HIV disclosure to their children using a standard Parental Disclosure Interview Questionnaire. Moreover, our finding generally agrees with observations from other researchers indicating higher rates of parental disclosure in developed countries compared to resource limited setting. For example a review by Clifford and colleagues documents disclosure rates ranging from 20-97 % in developed compared to 11- 44 % in Low and Middle Income Countries [15].In yet another review, Qiao and others report disclosure rates of 20 to 67 % for studies conducted in the United States of America and a figure as low as 11 % in some European countries [11]. Low disclosure rates in resource limited settings such as Uganda might signal differences in socio-cultural and family dynamics between developed and developing countries which makes HIV disclosure such a complicated social issue especially in resource limited settings. Indeed, it has been recognized that in order to promote parental HIV disclosure, more so in developing countries, there is need to develop culturally appropriate interventions. Where such interventions have been piloted, disclosure rates have markedly improved [16, 17] and this clearly underscores the importance of having many of such studies particularly in sub-Saharan Africa which continues to bare a huge burden of HIV/AIDS.

### Barriers to parental disclosure of the HIV status to children

From this study we found that almost half of parents who had not disclosed feared that children may tell other people about their HIV infection. The same fears have been documented in studies conducted in Botswana [18], Burkina Faso [19], South Africa [20] and in the United States of America [6]. However, such fears may reflect parents’ underestimate of the ability of children to keep family secrets. A study conducted in China found that a high proportion of children preferred not to disclose parental HIV status to other and they also did not like to tell truth to others in the situation of having to talk about parental HIV [21]. We also found that about fifty five percent of the parents who had not disclosed to their children did so because they did not want to worry or upset the children. This concurs with findings from countries such as United States of America [9, 22]. Another important barrier to disclosure was the perception by parents that children may not be able to understand what it means to be HIV positive. This finding agrees with what is reported in studies conducted in other countries [23, 24]. The age and indeed developmental stage of children are some of the key predicators of parental disclosure of their HIV sero-status such that disclosure rates tend to increase as children grow older.

### Factors associated with parental disclosure of their HIV status to children

One of the factors found to be positively associated with parental disclosure of their HIV status to their children is age of the parent. In this study we observed that increasing age of the parent was accompanied by increasing odds of disclosure. In other words as the parents get older, they are more likely to disclose their HIV status to their children. This concurs with findings from studies conducted in the United States of America [25], Burkina Faso [19] and China [26]. The influence of age on parental disclosure may be because as one lives longer with the disease, it becomes increasingly difficult to keep a positive HIV status secret. One way or the other the individual is forced sometimes by prevailing circumstances to disclose and indeed situations when disclosure occurred involuntarily are fairly common [27].

This study also revealed that parents who had gained tertiary level of education were less likely to disclose their HIV status to their children. This was a rather surprising finding because it would be expected that as it is with disclosure to sexual partners [28], attainment of higher education would be associated with a better understanding of the benefits of disclosure and therefore increasing the likelihood of parents disclosing their HIV status to their children. However, fear of lose of social standing which may result from children telling others about parent’s HIV status may present a significant barrier to disclosure thus downplaying the effect of education. Another unique finding in this study was that parents who belonged to the Pentecostal faith were more likely to disclose their HIV status to their children compared to parents who belonged to the protestant religious affiliation. The influence of religion on adherence to HIV treatment and care has been documented [29, 30]. However, it appears that in addition to adherence, religious affiliation, particularly being a Pentecostal has an influence HIV disclosure. This association merits further investigation especially given the fact the vast majority of Ugandans belong to one religion or another.

Another interesting find in this study was that parents who had someone make a commitment about the care of their children were more than twice likely to disclose their HIV status to at least one child compared to those parents who had not secured commitments from other people about the care of their children. Custodial planning has been cited as one of the reasons for maternal disclosure of HIV positive status [31]. In the Ugandan setting when a parent dies the children are usually taken care by the member of the extended family and most often than not, the parents of the diseased, sisters, or brothers assume the parenting responsibility. In preparation for transfer of such roles, it is not difficult to imagine that concerned individuals engage in open discussions in matters related to health and it is in such situations that disclosure to family members including children occurs. Just like any other studies, this study is not without limitations. The cross-sectional design used means that we cannot infer cause and effect relationships. Also the study relied on self- reported data which has a potential of introducing bias. However, we emphasized to the research participants the importance of honesty and truth telling. We also did not relate disclosure to the age or HIV status of the child which is a significant limitation in this study. Our findings also do not report on clinical condition of the clients and time since diagnosis both of which may have an impact on disclosure. Nevertheless, this study had important strengths. First, we had a reasonably large sample size as opposed to earlier studies whose findings were based on small samples Secondly, though the tool used was not locally validated, the fact that it was developed specifically for HIV infected parents is strength for this study.

## Conclusions

Parental disclosure of their HIV positive status to their children is still low in urban Kampala. Factors found to be associated with parental disclosure of the HIV positive status to their children were age of the parent, level of education, religion, and commitment from someone to care for children. If indeed by increasing parental disclosure of their HIV positive status to their children and by promoting open and honest discussions about HIV/AIDS at family level we hope to add one more weapon in the fight against HIV/AIDS, then there is need to develop locally relevant interventions to increase rates of disclosure.

## Abbreviations

AIDS, Acquired Immune Deficiency Syndrome; ART, Antiretroviral Therapy; HIV, Human immune deficiency virus; IDI, Infectious disease institute; IRB, Institutional review board; UNCST, Uganda national council for science and technology

## Additional file

Additional file 1: Disclosure data set. Parental disclosure of their HIV status to children. The data set contains information on all the variables investigated in this study. (XLS 647 kb)

## References

[1] Ministry of Health. The Uganda AIDS Indicator Survey. 2011.

[2] Corona R, Beckett MK, Cowgill BO, Elliott MN, Murphy DA, Zhou AJ, Schuster MA (2006). Do children know their parent’s HIV status? Parental reports of child awareness in a nationally representative sample. Ambul Pediatr.

[3] Moneyham L, Seals B, Demi A, Sowell R, Cohen L, Guillory J (1996). Experiences of disclosure in women infected with HIV. Health Care Women Int.

[4] Rwemisisi J, Wolff B, Coutinho A, Grosskurth H, Whitworth J (2008). ‘What if they ask how I got it?’ Dilemmas of disclosing parental HIV status and testing children for HIV in Uganda. Health Policy Plan.

[5] Lee MB, Rotheram-Borus MJ (2002). Parents’ disclosure of HIV to their children. Aids.

[6] Murphy DA, Steers WN, Dello Stritto ME (2001). Maternal disclosure of mothers’ HIV serostatus to their young children. J Fam Psychol.

[7] Murphy DA, Marelich WD, Amaro H (2009). Maternal HIV/AIDS and adolescent depression: A covariance structure analysis of the “Parents and Adolescents Coping Together” (PACT) model. Vulnerable Child Youth Stud.

[8] Mellins CA, Brackis-Cott E, Dolezal C, Leu CS, Valentin C, Meyer-Bahlburg HF (2008). Mental health of early adolescents from high-risk neighborhoods: the role of maternal HIV and other contextual, self-regulation, and family factors. J Pediatr Psychol.

[9] Delaney RO, Serovich JM, Lim JY (2008). Reasons for and against maternal HIV disclosure to children and perceived child reaction. AIDS Care.

[10] Wiener LS, Battles HB, Heilman NE (1998). Factors associated with parents’ decision to disclose their HIV diagnosis to their children. Child Welfare.

[11] Qiao S, Li X, Stanton B (2013). Disclosure of parental HIV infection to children: a systematic review of global literature. AIDS Behav.

[12] Institute ID. Infectious Diseases Institute Annual report; Infectious Diseases Institute, College of Health sciencesMakerere University; Available: www.idi-makerere.com. 2014.

[13] Pilowsky DJ, Sohler N, Susser E (1999). The parent disclosure interview. AIDS Care.

[14] Armistead L, Tannenbaum L, Forehand R, Morse E, Morse P (2001). Disclosing HIV status: are mothers telling their children?. J Pediatr Psychol.

[15] Clifford G, Craig GM, McCourt C, Barrow G (2013). What are the benefits and barriers of communicating parental HIV status to seronegative children and the implications for Jamaica? A narrative review of the literature in low/middle income countries. West Indian Med J.

[16] Rochat TJ, Mkwanazi N, Bland R (2013). Maternal HIV disclosure to HIV-uninfected children in rural South Africa: a pilot study of a family-based intervention. BMC Public Health.

[17] Rochat TJ, Arteche AX, Stein A, Mkwanazi N, Bland RM (2014). Maternal HIV disclosure to young HIV-uninfected children: an evaluation of a family-centred intervention in South Africa. AIDS.

[18] Nam SL, Fielding K, Avalos A, Gaolathe T, Dickinson D, Geissler PW (2009). Discussing matters of sexual health with children: what issues relating to disclosure of parental HIV status reveal. AIDS Care.

[19] Tiendrebeogo G, Hejoaka F, Belem EM, Compaore PL, Wolmarans L, Soubeiga A, Ouangraoua N (2013). Parental HIV disclosure in Burkina Faso: experiences and challenges in the era of HAART. SAHARA J.

[20] Madiba S (2013). The impact of fear, secrecy, and stigma on parental disclosure of HIV status to children: a qualitative exploration with HIV positive parents attending an ART clinic in South Africa. Glob J Health Sci.

[21] Qiao S, Li X, Zhao G, Zhao J, Stanton B (2012). Secondary disclosure of parental HIV status among children affected by AIDS in Henan, China. AIDS Patient Care STDS.

[22] Kennedy DP, Cowgill BO, Bogart LM, Corona R, Ryan GW, Murphy DA, Nguyen T, Schuster MA (2010). Parents’ disclosure of their HIV infection to their children in the context of the family. AIDS Behav.

[23] Gachanja G, Burkholder GJ, Ferraro A (2014). HIV-positive parents, HIV-positive children, and HIV-negative children’s perspectives on disclosure of a parent’s and child’s illness in Kenya. PeerJ.

[24] Zhang L, Li X, Zhao J, Zhao G, Kaljee L, Stanton B (2013). Disclosure of parental HIV infection to children and psychosocial impact on children in China: a qualitative study. Asia Pac J Couns Psychother.

[25] Letteney S, Krauss B, Kaplan R (2012). Examining HIV-positive parents’ disclosure to their children: a biopsychosocial approach. Soc Work Public Health.

[26] Zhou Y, Zhang L, Li X, Kaljee L (2013). Do Chinese parents with HIV tell their children the truth? A qualitative preliminary study of parental HIV disclosure in China. Child Care Health Dev.

[27] Varga CA, Sherman GG, Jones SA (2006). HIV-disclosure in the context of vertical transmission: HIV-positive mothers in Johannesburg. South Africa AIDS Care.

[28] Batte A, Katahoire AR, Chimoyi A, Ajambo S, Tibingana B, Banura C (2015). Disclosure of HIV test results by women to their partners following antenatal HIV testing: a population-based cross-sectional survey among slum dwellers in Kampala Uganda. BMC Public Health.

[29] Kisenyi RN, Muliira JK, Ayebare E (2013). Religiosity and adherence to antiretroviral therapy among patients attending a public hospital-based HIV/AIDS clinic in Uganda. J Relig Health.

[30] Tumwine C, Neema S, Wagner G (2012). Reasons Why High Religiosity Can Co-exist with and Precipitate Discontinuation of Anti-retroviral Therapy among Different HIV Clients in Uganda: An Exploratory Study. Religions (Basel).

[31] Pilowsky DJ, Sohler N, Susser E (2000). Reasons given for disclosure of maternal HIV status to children. J Urban Health.

